# Bioinspired Fabrication of an Insensitive Ammonium Perchlorate Core–Shell Composite with Polydopamine Coating

**DOI:** 10.3390/polym16081069

**Published:** 2024-04-11

**Authors:** Yafeng Huang, Xuan Tian, Jinfei Wang, Kejun Zhong, Yuan Chen, Chenglong Li, Pengxiang Jia

**Affiliations:** 1 Xi’an Modern Chemistry Research Institute, Xi’an 710065, China; huangyafeng204@outlook.com (Y.H.); aaaxuanyuan@outlook.com (X.T.); 2 Key Laboratory of Synthetic and Natural Functional Molecule of Ministry of Education, College of Chemistry and Materials Science, Northwest University, Xi’an 710127, China; wjf152326@outlook.com (J.W.); zhongkejun@stumail.nwu.edu.cn (K.Z.); chenyuansky@outlook.com (Y.C.); lichenglong@stumail.nwu.edu.cn (C.L.)

**Keywords:** ammonium perchlorate, polydopamine, mechanical sensitivity

## Abstract

In this research, an ammonium perchlorate/polydopamine (AP/PDA) core–shell composite was prepared in a non-aqueous solution to reduce the mechanical sensitivity of ammonium perchlorate (AP). The result showed that the AP/PDA core–shell composite could be successfully constructed in ethyl acetate solution with an AP recovery rate that reached 86%. The mechanical sensitivity of the obtained AP/PDA core–shell composite was significantly reduced with a PDA content of only 0.76%. The DSC and TG also indicated that the coating of PDA showed catalytic activity in the thermal decomposition of AP with a lower decomposition temperature and a decreased E_a_ value of AP. Thus, this study proposed a simple strategy for achieving a good balanced between harnessing the energy and ensuring the safety of ammonium perchlorate by significantly reducing its mechanical sensitivity by using a very low polydopamine coating layer content, and this shows great potential for the design and fabrication of insensitive energetic composites for use in propellants.

## 1. Introduction

Ammonium perchlorate (AP) has been widely used as a solid propellant due to the advantage of its high energy content [1]. It is well known that a high burning rate of AP based solid propellants is desirable to improve the range of solid rockets. Previous studies showed that the improvement of the burning rate could be achieved by using a decomposition catalyst for AP, such as metallic compounds, metal–organic frameworks and so on. For example, Hu and co-workers prepared hollow mesoporous CuO microspheres using the hard-template method and then applied it in catalyzing AP decomposition [2]. It was found that the hollow mesoporous CuO microspheres could decrease the AP decomposition temperature by 105.7 °C and enhance the heat production by 735 J/g. Yang and co-workers prepared a metal–organic framework (MOF) material, Fe-benzene-1,3,5-tricarboxylate (Fe-BTC), and then used it as a combustion catalyst for solid propellants containing ammonium perchlorate [3]. The thermal decomposition of ammonium perchlorate (AP) was evidently found to be enhanced in the presence of Fe-BTC, resulting in a decomposition temperature by 36 °C. Zhang and colleagues synthesized a novel graphene–ferrocene nanocomposite (G-792-Fe) and used it as a multifunctional combustion catalyst in an ammonium perchlorate-hydroxyl terminated polybutadiene (AP-HTPB) propellant [4]. The G-792-Fe not only showed an excellent combustion catalytic performance on the AP-HTPB propellant (in which the burning rate of the AP-HTPB propellant containing G-792-Fe increased from 13.87 mm·s^−1^ to 17.28 mm·s^−1^ at 15 MPa), but also an improvement in the anti-migration performance, safety performance and mechanical properties of the AP-HTPB propellant. Unfortunately, adding a catalyst would decrease the energy density of AP. Enhancing the burning rate of AP could also be realized by decreasing the particle size of AP, owing to the fast reaction rate caused by the enhanced specific surface area and the reduction in the diffusion distance [5,6]. For example, Kohga found that the relationship between the burning rate and the weight mean diameter (Dw) is divided into two regions. The burning rate was almost constant above the critical Dw and increased with the decreasing Dw below that [7]. Sunil and co-workers also found that, as size of the AP decreases, the propellant slurry viscosity increases and the burn rate increases [8]. However, a decrease in the particle size would also cause an elevation of the mechanical sensitivity of AP [9]. Therefore, it is still a challenge to balance the contradiction that occurs among high energies, high burning rates and low mechanical sensitivities.

In recent decades, various strategies have been proposed to decrease the mechanical sensitivity of AP. Generally speaking, the most common methods for the desensitization of AP are periodization, the fabrication of energetic composites and surface coatings [10]. Periodization can decrease the mechanical sensitivity of AP because the crystal defects within AP are avoided by spheroidizing the particles [11,12,13]. For example, Song and co-workers prepared superfine spherical AP particles from smashed ammonium perchlorate with a jet mill, and then milled the particles with a planetary ball mill [11]. They also found that the impact sensitivity and friction sensitivity of superfine spherical AP are lower than the non-spherical AP, and hygroscopicity and caking are effectively improved. Wan and co-workers prepared spherical AP particles by forward-reverse rotation using a vertical grinding mill [12]. They found that the impact sensitivity and friction sensitivity of spherical AP particles were decreased by 32% and 22%, respectively, compared with the non-spherical AP with the same particle size. However, the sensitivity-reducing effect of this method always has limitations. The desensitization of AP can also be achieved by mixing it with fillers with a low mechanical sensitivity, such as metal compounds, carbon materials and so on [14,15]. Cheng and co-workers synthesized energetic graphene oxide (GO)-based burning rate catalysts (FGO 1–6) with excellent desensitization performances for ammonium perchlorate (AP) through a nucleophilic substitution reaction of energetic functional groups with acylated GO. They found that the impact and friction sensitivities of the AP/FGO mixtures clearly decreased in comparison with pure AP. When the mass ratio of FGO-6 was 2.5 mass%, the impact and friction sensitivities of AP reduced from 8.0 to 12.3 J and 120 to 218 N, respectively [16]. Liu and colleagues prepared an ammonium perchlorate-based molecular perovskite energetic material, (H_2_dabco)[NH_4_(ClO_4_)_3_]/carbon nanotubes (DAP/CNTs) [17]. The results showed that the mechanical sensitivity (impact and friction sensitivities: >120 cm and 20%) and electrostatic spark sensitivity (8.90 J) of the DAP/CNTs energetic composite with 10 wt.% CNTs exhibited less sensitivity than DAP (impact, friction and electrostatic spark sensitivities: 112.3 cm, 45% and 5.39 J, respectively), because of the mixing desensitization mechanism of the CNTs. But such methods are complex and time consuming, and they have not yet been applied in the processing of a propellant.

Surface coatings are frequently used for the desensitization of AP because they are simple, effective and applicable [18]. Various materials have been applied in surface coatings of AP, such as insensitive explosives, paraffin wax, carbon materials and polymers, to reduce its mechanical sensitivity [19,20]. Nandagopal and co-workers used fluorocarbon polymers (copolymer of hexafluoropropylene and vinylidene fluoride, HFP-VF) to reduce the mechanical sensitivity of AP [21]. It was found that the impact sensitivity decreases after increasing the percentage of HFP-VF-coated AP while no change is observed in the friction sensitivity value. Yang and co-workers applied several inert materials, including some functional carbon materials, paraffin wax and the well-known insensitive energetic material 1,3,5-triamino-2,4,6-trinitrobenzene (TATB), to reduce the undesirable high sensitivity of ultra-fine ammonium perchlorate (UF-AP) via a polymer-modified coating [22]. They found that graphene, graphene oxide and graphene nanosheets display an efficient desensitization of impact, while graphite and nTATB definitely decrease the friction sensitivity. The well-known passivation agent, wax, shows moderate desensitization for both impact and friction. However, the interfacial interactions between the coating layer and AP particles are usually very low because of the low adhesion properties of AP particles. Therefore, it is critical to reduce mechanical sensitivity by enhancing the interfacial interactions between the coating layers and AP crystals.

Recently, mussel-inspired polydopamine (PDA) coating leveraging the in situ self-polymerization of dopamine has attracted increasing interest due to the combined advantages of easy processing and high interfacial interactions with various materials, such as metals, woods, polymers, carbon materials and inorganic nonmetallic materials [23]. Polydopamine has been adopted for the coating of energetic materials, such as HMX, RDX, TATB and CL-20, to improve their stability [24,25,26,27]. He and co-workers prepared PDA-modified TATB (pTATB) with a compact core–shell structure through the self-polymerization of dopamine [28]. They found that the PBX containing pTATB exhibited significantly improved tensile and compression strength or strain, and creep resistance, due to the strong interfacial interactions within the PDA interlayer. Zhu and co-workers modified explosives (HMX@PDA, TATB@PDA) via the polymerization of dopamine [29]. It was found that the characteristic complete and compact coating of polydopamine endowed the modified explosives with a reduced sensitivity and improved interface mechanical properties and processing stability. Zhang and colleagues prepared ε-CL-20/polydopamine core–shell microcapsule particles [30]. The results showed that the compact and dense coating delayed the ε-CL-20 crystal transformation temperature by about 30 °C, which enhanced the particles’ thermal stability. In addition, with the coating via polydopamine, the friction sensitivity of ε-CL-20 crystals decreases significantly. However, to our knowledge, no research on the application of polydopamine coatings for the desensitization of AP has been reported. This is attributed to the high solubility of AP in water, making it unsuitable for traditional polydopamine coating conditions (Tris-HCl solution, pH 8.5).

In this work, an AP/PDA core–shell composite was prepared in a non-aqueous solution to reduce the mechanical sensitivity of AP. The result showed that the AP/polydopamine core–shell composite could be successfully constructed in an ethyl acetate solution with a high AP recovery rate. The mechanical sensitivity of AP was significantly reduced when the content of the polydopamine was only 0.76%. The DSC and TG also indicated that the coating of PDA showed catalytic activity in the thermal decomposition of AP.

## 2. Experimental Section

### 2.1. Materials

Ammonium perchlorate was obtained from Liming Chemical Research and Design Institute Co., Ltd. (Xi’an, China). Anhydrous ethanol, ethyl acetate, dopamine hydrochloride and diethylamine were purchased from Aladdin (Shanghai, China). All of the chemicals were used directly as received.

### 2.2. Preparation of the AP/PDA Core-Shell Composite

First, 200 mg dopamine hydrochloride was dissolved in 50 mL ethyl acetate and stirred until completely dissolved. Then, 4 g ammonium perchlorate was added to the above solution and stirred for 10 min. Next, 500 mL of diethylamine was dropped into the solution, followed by a reaction at 25 °C for 24 h. Then, the obtained dark AP solution was filtered. The filter cake was washed with ethyl acetate and then dried at 60 °C for 6 h to obtain the AP/PDA core–shell composite. The amount of dopamine hydrochloride was adjusted while other conditions were kept unchanged to obtain the optimal reaction conditions. The AP/PDA core–shell composite is denoted as AP/PDA_x_, where x represents the concentration of the dopamine hydrochloride (mg/mL). For the control group, anhydrous ethanol was used as the reaction medium.

### 2.3. Characterization

Raman tests were performed on an DXR2 Raman Microscopy (ThermoFisher, Waltham, MA, USA) with a scan range from 3500 to 500 cm^−1^. The excitation wavelength was 633 nm with power less than 10 mW. The Raman spectra were taken unpolarized. The morphology of AP and AP/PDA core–shell composite was observed by a SU8010 scanning electron microscopy (SEM, Ibaraki, Japan) at an accelerating voltage of 5 kV. X-ray photoelectron spectroscopy (XPS) was used for further investigations of the surface chemical compositions. And high-resolution spectra of O 1s, C 1s and N 1s were recorded and analyzed. The spectra were measured with a PHI 5000 VersaProbe III system (ULVAC-PHI, Chigasaki, Japan) using Mg Kα radiation (300 W, 1253.6 eV) with the air pressure of 3 × 10^−9^ mbar. All XPS spectra were collected at an electron takeoff angle of 70° from the surface. Binding energies were calibrated relative to the C 1s’ peak (284.8 eV) from hydrocarbons being adsorbed onto the surface of the samples. XRD curves were obtained using powder X-ray diffraction (XRD) on a PANalytical X’Pert Pro X-ray Diffractometer (Panalytical, Almelo, The Netherlands)with Cu Ka radiation (*λ* = 1.5418 Å) at 40 kV and 40 mA. X-ray diffraction patterns were taken from 5° to 50° (2*θ*) in steps of 0.05° at ambient temperature. Thermogravimetric analysis (TG) was performed on a simultaneous thermal analyzer instrument (SDTQ600, TA, New Castle, DE, USA), and each sample with a weight of 10–15 mg was heated from room temperature to 500 °C under a nitrogen atmosphere (purity above 99.99%) with a heating rate of 10 °C·min^−1^. The study of the thermal decomposition of AP and AP/PDA core–shell composite was also carried out using the DSC 200 F3 model (Netzsch, Hanau, Germany). In order to mitigate the transport phenomena, the sample mass was kept at ~10 mg in all of the cases. The samples were placed in closed Al_2_O_3_ pans with a small hole at the top and heated from room temperature to 500 °C. The heating rates were controlled at 5.0, 10.0 and 20.0 °C·min^−1^, respectively, under a nitrogen atmosphere (purity above 99.99%) with flow rate of 50 mL· min^−1^. The DSC curves were obtained with sampling rate of 12–20 points per centigrade in non-isothermal mode, which is sufficient to eliminate errors in the evaluation of both enthalpy and kinetic parameters.

### 2.4. Mechanical Sensitivity Tests

The impact sensitivity was tested using a WL-1 type impact sensitivity apparatus based on a measurement standard of GJB-772A-1997 [31] with a 10 kg drop weight and sample masses of 50 mg. The impact sensitivity was recorded according to the special height (H_50_) value, which represented the drop height with a 50% explosion probability in 50 tests. A friction sensitivity test was conducted using an MGY-1 type friction sensitivity instrument based on a measurement standard of GJB-772A-1997 with a sample mass of 20 mg, a pendulum weight of 1.5 kg and a pendulum angle of 90°. The fraction sensitivity was expressed by the explosion probability (P) from 50 tests.

## 3. Results and Discussions

### 3.1. Preparation of the AP/PDA Core-Shell Composite

**Figure 1:**
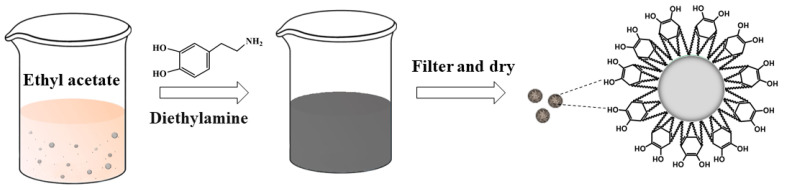
Preparation of the AP/PDA core–shell composite.

**Figure 2:**
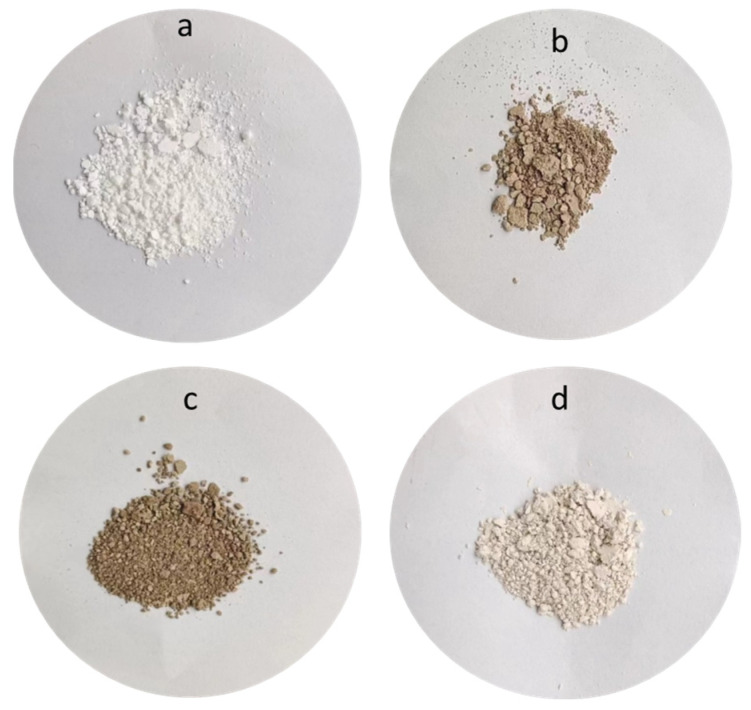
Pictures of the AP/PDA core–shell composites: (**a**) AP, (**b**) AP/PDA_1_, (**c**) AP/PDA_4_, (**d**) AP/PDA_4_ in ethanol.

As shown in Figure 1, an AP/PDA core–shell composite was fabricated at room temperature via the self-polymerization of polydopamine using diethylamine as the catalyst. After reacting for about 6 h, the white suspension of AP turned brown. After reacting for 12 h, the color became dark. Pictures of the AP/PDA core–shell composites are shown in Figure 2. The white AP powder became black after being coated with polydopamine. The results also showed that the AP/PDA_4_ sample, which was prepared in ethyl acetate with a dopamine concentration of 4.0 mg/mL, had the deepest color. These results strongly suggested that a polydopamine layer was successfully coated onto the surface of the AP particles.

The PDA content and AP recovery rate of the AP/PDA composites are shown in Table 1. The results indicated that the AP/PDA composites fabricated in ethyl acetate had a much higher relative PDA content and AP recovery rate than those of the samples fabricated in ethanol. The reason was ascribed to the high solubility of AP in ethanol. It was also found that the AP/PDA_4_ sample displayed a large PDA content and high AP recovery rate, so it was chosen for the following experiments.

**Figure 3:**
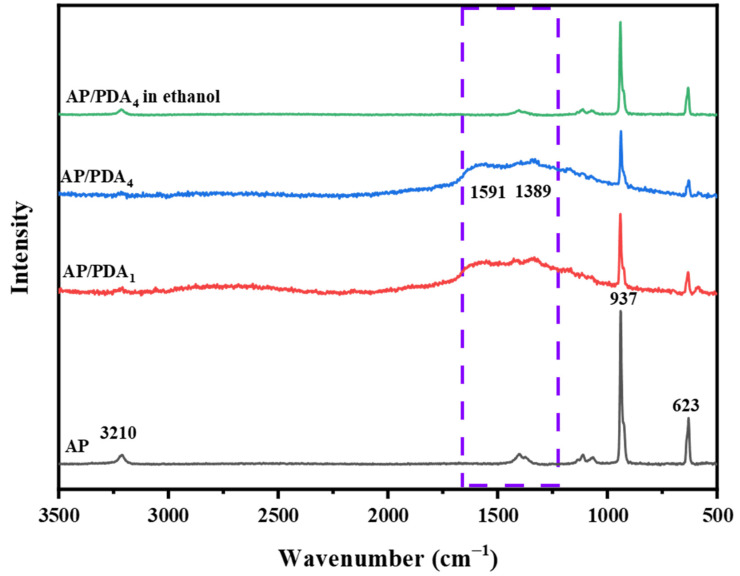
Raman spectra of the AP/polydopamine core–shell composites.

The Raman spectra of the AP/polydopamine core–shell composites are demonstrated in Figure 3. The typical adsorption bands of AP were located at 3210, 937 and 623 cm^−1^. The peaks at 3210 cm^−1^were ascribed to the NH_4_ stretching vibration, while the bands at 937 and 623 cm^−1^ were ascribed to the stretching vibrations of ClO_4_ [32]. After coating with polydopamine, two new peaks located at 1591 and 1389 cm^−1^, corresponding to PDA, appeared. The peak at 1591 cm^−1^ arose from the C=C aromatic ring vibration, and the peak at 1389 cm^−1^ was attributed to the aromatic C–N stretching mode of the indole structure of PDA [33]. The results indicated the successful coating of a polydopamine layer onto the surfaces of the AP particles.

The surface element compositions of AP and the AP/PDA core–shell composites were carefully studied via XPS, and the results are shown in Table 2. Compared with AP, the AP/PDA core–shell composites showed much larger N and O contents. It was found that AP had an N/O atomic ratio of 0.17, which was close to the theoretical value of AP (0.22). After coating with PDA, the N/O atomic ratios of AP/PDA_1_, AP/PDA_4_ and AP/PDA_4_ in ethanol were increased to 0.31, 0.35 and 0.28, respectively, due to the large N/O atomic ratio of PDA (the theoretical value was 0.44). These results indicated the successful coating of PDA onto the surfaces of the AP particles.

**Figure 4:**
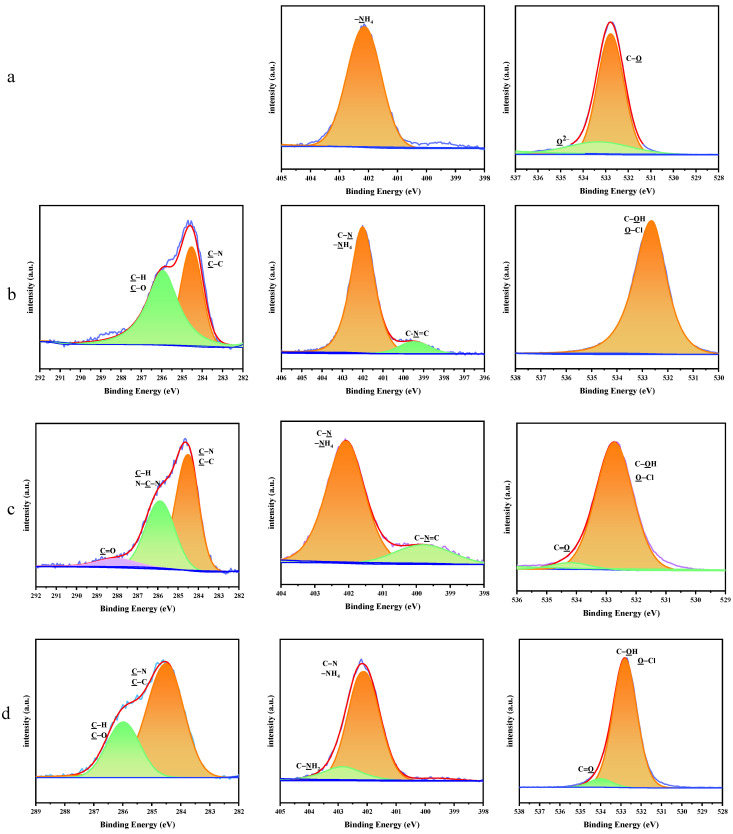
High−resolution XPS spectra of the C 1s, N 1s and O 1s peaks for (**a**) AP, (**b**) AP/PDA_1_, (**c**) AP/PDA_4_, and (**d**) AP/PDA_4_ in ethanol.

The high-resolution XPS spectra of the C 1s, N 1s and O 1s peaks for AP and the AP/PDA core–shell composites are shown in Figure 4. For AP, the N 1s peak was fitted to one peak, which was ascribed to –NH_4_. The Cl–O peak was also clearly observed in the fitted peaks of O 1s. After coating with PDA, the characteristic peaks of PDA appeared in the XPS spectra of the AP/PDA core–shell composites (C–O, C=O, C–N, C–C in the C 1s, C-N, C–N=C in N 1s and C=O in O 1s). These results further confirmed the successful coating of PDA onto the surfaces of the AP particles.

**Figure 5:**
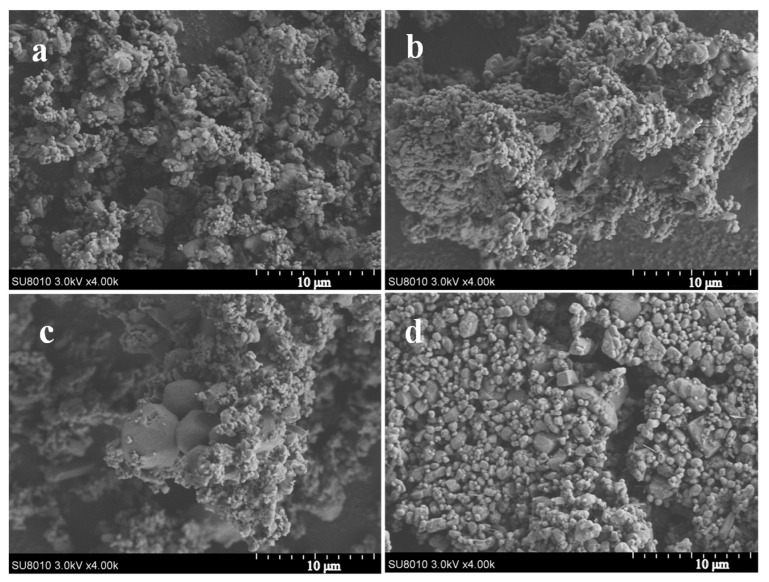
SEM images of the AP/PDA core–shell composites, (**a**) AP, (**b**) AP/PDA_1_, (**c**) AP/PDA_4_, (**d**) AP/PDA_4_, in ethanol.

SEM images of the AP and AP/PDA core–shell composites are shown in Figure 5. It was found that the morphology of the AP and AP/PDA composites showed no large difference when the PDA coating was fabricated in ethyl acetate. The reason was attributed to the low PDA content of the AP/PDA composites, and the thin PDA coating layer had no obvious effects on the morphology of the AP particles. In contrast, when the AP/PDA composite was fabricated in ethanol, small AP particles were not observed, indicating the partial dissolution of AP particles in ethanol.

**Figure 6:**
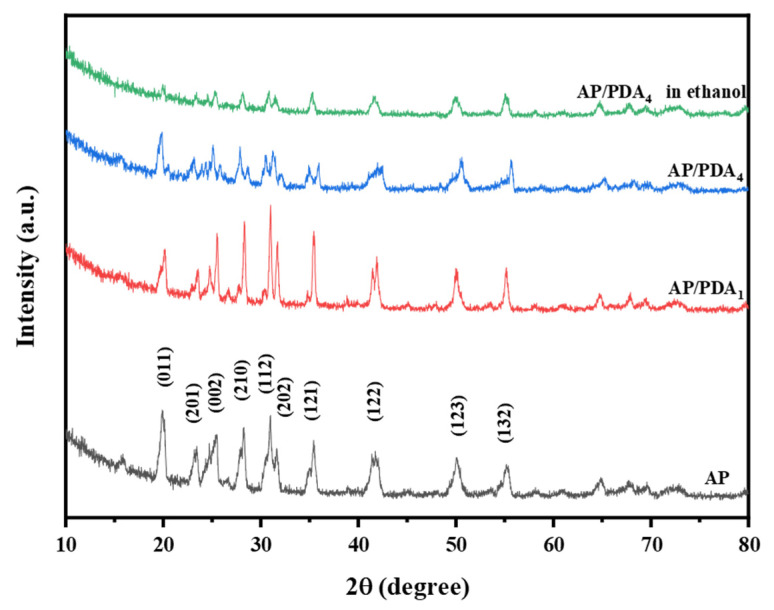
XRD patterns of AP and the AP/PDA core–shell composites.

The XRD patterns of AP and the AP/PDA core–shell composites are displayed in Figure 6. The typical peaks of AP/PDA_1_ and AP/PDA_4_ were entirely consistent with the characteristic peaks of AP, suggesting that the crystal structure of AP was not changed after being coated with PDA. The reason could be attributed to the low content and amorphous structure of PDA. For AP/PDA_4_ in ethanol, the diffraction peak intensity remarkably decreased, and the typical (011) peak at 20° almost disappeared, indicating that the crystal structure of AP was changed due to the partial dissolution of AP in ethanol.

### 3.2. Thermal Decomposition Properties of the AP/PDA Core-Shell Composite

**Figure 7:**
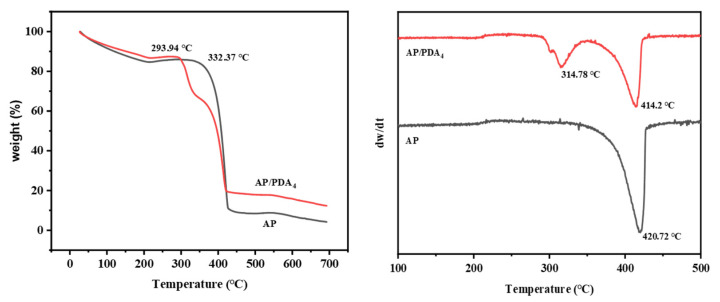
TG and DTG curves of AP and AP/PDA core–shell composite.

The thermal decomposition properties of AP and the AP/PDA core–shell composites were studied with TG (Figure 7). The decomposition of AP mainly happened at temperatures ranging from 330 °C to 430 °C. After being coated with PDA, AP/PDA_4_ indicated a two-stage process of weight loss: the first weight loss started when the temperature reached about 290 °C, and it was followed by a second weight loss when the temperature exceeded 330 °C. In our previous work, we found that the decomposition of PDA happened in a wide temperature range from 200 to 600 °C [34]. Thus, the first weight loss in AP/PDA_4_ was mainly caused by the decomposition of PDA, while the second weight loss was mainly caused by the decomposition of AP. A decrease in the peak decomposition temperature of the AP component from 420.7 to 414.2 °C compared with that of pure AP was observed. These results indicated that the presence of PDA could accelerate the decomposition of AP.

**Figure 8:**
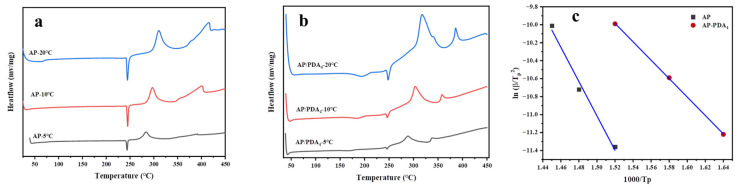
DSC curves of AP (**a**) and AP/PDA core–shell composite (**b**). Fitting curve of ln(β/T_p_^2^) against 1000/Tp (**c**).

The DSC curves of AP (a) and the AP/PDA core–shell composite and the corresponding fitting curves of ln(β/Tp^2^) against 1000/Tp are shown in Figure 8. The decomposition of AP and the AP/PDA core–shell composites could be divided into three stages: the first peaks located at 248 °C were ascribed to the crystal phase transition of AP, followed by the low-temperature decomposition peak and high-temperature decomposition peak. The high-temperature decomposition peaks of AP and the AP/PDA core–shell composites at different heating rates are shown in Table 3. The apparent activation energy (E_a_) values of AP and the AP/PDA core–shell composites were calculated using the Kissinger equation [35]:

$$\ln\left(\frac{\beta }{T_{p}^{2}}\right)=-\frac{E_{a}}{{\mathrm{RT}}_{p}}+\ln\left(\frac{\mathrm{AR}}{E_{a}}\right)$$

where, β, T_p_, E_a_, A and R represent the heating rate, the high-temperature decomposition peak at the differential heating rate, the apparent activation energy, the preexponential factor and the universal gas constant, respectively. As shown in Figure 8c and Table 3, the results indicated that the AP/PDA core–shell composites had much a lower apparent activation energy (84.4 KJ∙mol^−1^) than that of AP (175.5 KJ∙mol^−1^). Thus, the DSC and TG experiments indicated that the coating of PDA showed catalytic activity in the thermal decomposition of AP by lowering the decomposition temperature and decreasing the E_a_ value of AP.

### 3.3. Mechanical Sensitivity of the AP/PDA Core-Shell Composites

The mechanical sensitivity of the AP/PDA core–shell composites was carefully investigated, as shown in Table 4. The impact sensitivity of AP was greatly decreased after being coated with PDA. For example, the original AP showed the lowest H_50_ of 69.6 cm, while this value was greatly improved to 125.9 cm when the AP was coated in a 4.0 mg/mL dopamine solution for 24 h. It was found that the dopamine concentration had an important effect on the impact sensitivity of the AP/PDA core–shell composites. When the concentration of the dopamine solution increased from 1.0 mg/mL to 4.0 mg/mL, a remarkable enhancement of the H_50_ value from 85.4 cm to 125.9 cm was observed. The results also suggested that the reaction medium also had a large effect on the impact sensitivity: when the reaction medium was ethanol, a slight increase in H_50_ from 69.6 cm to 79.5 cm was observed, although the concentration of dopamine was the same as that in ethyl acetate. More importantly, in ethanol, the AP/PDA_4_ sample showed a poorer impact sensitivity than the AP/PDA_1_ sample despite it having a much larger PDA content (0.56%, while PDA content of AP/PDA1 was 0.34). These results strongly suggested that the AP solubility of the coating medium had a critical effect on the mechanical sensitivity of the AP/PDA core–shell composites. The reason for this might be ascribed to the formation of an irregular shape due to the partial dissolution of AP particles in ethanol. The friction sensitivity also showed a similar tendency: the friction sensitivity of AP was largely decreased after being coated with PDA, and the AP/PDA_4_ sample showed the lowest friction sensitivity when the reaction was conducted in ethyl acetate with a dopamine concentration of 4.0 mg/mL. The remarkable decrease in the impact sensitivity and friction sensitivity of AP via the coating with PDA might be attributed to the large interfacial interaction between AP and PDA, and the uniform and compact PDA layer could help to optimize the crystal defects within the AP particles.

## 4. Conclusions

AP/PDA core–shell composites were prepared in a non-aqueous solution to reduce the mechanical sensitivity of AP. The results showed that AP/PDA_4_ sample, which was constructed in ethyl acetate with a dopamine concentration of 4.0 mg/mL, showed the optimal coating effect and had an AP recovery rate that reached 86%. The results exhibited that the impact sensitivity (>125.9 cm) and friction sensitivity (8%) of the AP/PDA_4_ composite were significantly reduced compared with those of pure AP (impact sensitivity: 69.6 cm, friction sensitivity: 76%) and the PDA content was only 0.76%. The results also showed that the AP solubility of the coating medium had critical effect on the mechanical sensitivity of the AP/PDA core–shell composites.

The DSC and TG also indicated that the coating of PDA showed catalytic activity in the thermal decomposition of AP by lowering the decomposition temperature and decreasing the E_a_ value of AP. Thus, this study proposed a simple strategy for achieving a good balance between harnessing the energy and ensuring the safety of ammonium perchlorate by significantly reducing its mechanical sensitivity with a very low polydopamine coating content. The method presented in this work could be expanded for the desensitization of other water-soluble energetic materials.

## Figures and Tables

**Table 1:** Parameters of the AP/PDA composites.

Sample	PDA Content (%)	AP Recovery Rate (%)
AP/PDA_1_	0.34	87.2%
AP/PDA_3_	0.67	86.9%
AP/PDA_4_	0.76	86.7%
AP/PDA_5_	0.47	86.4%
AP/PDA_4_ in ethanol	0.56	31.2%

**Table 2:** The surface element compositions of AP and the AP/PDA core–shell composites.

Sample	C 1s (%)	N 1s (%)	O 1s (%)	Cl 2p (%)
AP	50.53	6.16	37.29	6.02
AP/PDA_1_	15.36	18.44	59.16	7.04
AP/PDA_4_	30.85	16.91	48.83	3.41
AP/PDA_4_ in ethanol	6.15	20.24	72.17	1.44

**Table 3:** Apparent activation energy values of AP and the AP/PDA core–shell composites.

Sample	β/(K∙min^−1^)	T_p_/°C	Ea/(KJ∙mol^−1^)	R^2^
AP	5	381.4	175.5	0.9875
	10	400.5		
	20	415.5		
AP/PDA_4_	5	337.4	84.4	0.9998
	10	358.1		
	20	386.1		

**Table 4:** Impact and friction sensitivity of AP and the AP/PDA composites.

Sample	Impact Sensitivity (H_50_, cm)	Friction Sensitivity (P, %)
AP	69.6	76
AP/PDA_1_	85.4	24
AP/PDA_4_	≥125.9	8
AP/PDA_4_ in ethanol	79.5	24

## Data Availability

Data are contained within the article.
